# The Complete Chloroplast Genome of a Key Ancestor of Modern Roses, *Rosa chinensis* var. *spontanea*, and a Comparison with Congeneric Species

**DOI:** 10.3390/molecules23020389

**Published:** 2018-02-12

**Authors:** Hong-Ying Jian, Yong-Hong Zhang, Hui-Jun Yan, Xian-Qin Qiu, Qi-Gang Wang, Shu-Bin Li, Shu-Dong Zhang

**Affiliations:** 1 National Engineering Research Center for Ornamental Horticulture/Flower Research Institute, Yunnan Academy of Agricultural Sciences, Kunming 650205, China; hjyan8203@126.com (H.-J.Y.); xianqin711@sina.com (X.-Q.Q.); wqg712@sina.com (Q.-G.W.); sbli315@126.com (S.-B.L.); 2 School of Life Sciences, Yunnan Normal University, Kunming 650500, China; yhzhang@mail.kib.ac.cn; 3 School of Biological Sciences and Technology, Liupanshui Normal University, Liupanshui 553004, China

**Keywords:** *Rosa chinensis* var. *spontanea*, chloroplast genome, repeats, SSRs, genome comparison, phylogeny

## Abstract

*Rosa chinensis* var. *spontanea*, an endemic and endangered plant of China, is one of the key ancestors of modern roses and a source for famous traditional Chinese medicines against female diseases, such as irregular menses and dysmenorrhea. In this study, the complete chloroplast (cp) genome of *R. chinensis* var. *spontanea* was sequenced, analyzed, and compared to congeneric species. The cp genome of *R. chinensis* var. *spontanea* is a typical quadripartite circular molecule of 156,590 bp in length, including one large single copy (LSC) region of 85,910 bp and one small single copy (SSC) region of 18,762 bp, separated by two inverted repeat (IR) regions of 25,959 bp. The GC content of the whole genome is 37.2%, while that of LSC, SSC, and IR is 42.8%, 35.2% and 31.2%, respectively. The genome encodes 129 genes, including 84 protein-coding genes (PCGs), 37 transfer RNA (tRNA) genes, and eight ribosomal RNA (rRNA) genes. Seventeen genes in the IR regions were found to be duplicated. Thirty-three forward and five inverted repeats were detected in the cp genome of *R. chinensis* var. *spontanea.* The genome is rich in SSRs. In total, 85 SSRs were detected. A genome comparison revealed that IR contraction might be the reason for the relatively smaller cp genome size of *R. chinensis* var. *spontanea* compared to other congeneric species. Sequence analysis revealed that the LSC and SSC regions were more divergent than the IR regions within the genus *Rosa* and that a higher divergence occurred in non-coding regions than in coding regions. A phylogenetic analysis showed that the sampled species of the genus *Rosa* formed a monophyletic clade and that *R. chinensis* var. s*pontanea* shared a more recent ancestor with *R. lichiangensis* of the section *Synstylae* than with *R. odorata* var. *gigantea* of the section *Chinenses*. This information will be useful for the conservation genetics of *R. chinensis* var. *spontanea* and for the phylogenetic study of the genus *Rosa*, and it might also facilitate the genetics and breeding of modern roses.

## 1. Introduction

Molecular data have suggested that *Rosa chinensis* Jacq. var. *spontanea (*Rehder et*.* Wilson) Yü et Ku is the maternal parent of *R. chinensis* and the possible paternal parent of *R. odorata* (Andrews) Sweet [1], which gave characters of recurrent flowering, tea scent, and multiple floral colors to modern roses [2,3]. As one of the key ancestors of modern roses, *R. chinensis* var. *spontanea* is not only a precious germplasm resource for improving modern roses but also valuable plant material for the genetic research of recurrent flowering and the study of the biosynthesis of flower scent components. Furthermore, with the effect of “promoting blood circulation for removing blood stasis” and “subdhing swelling and detoxicating”, *R. chinensis* var. *spontanea* is also a source for famous traditional Chinese medicines that treat female diseases such as irregular menses and dysmenorrhea [4].

*Rosa chinensis* var. *spontanea* originates from China and is endemic to the Hubei, Sichuan, Chongqing, and Guizhou provinces [5,6]. It has been overharvested by local people and pharmaceutical companies because of its medicinal usefulness and has become rare in its wild habitats. It was uncertain whether it still existed as a wild-living species because investigators failed to collect samples of this species in the field [1]. It has been listed as an endangered (EN) species in a recent biodiversity report [7]. Fortunately, during systematic and integrative field investigations focusing on this species, we recently found several populations in the wild.

It is important to mention that little information is available about *R. chinensis* var. *spontanea* except the fact that it is a diploid plant [8] and that it emits 1,3,5-trimethoxybenzene, together with methyleugenol and isomethyleugenol, as minor floral scent compounds [9], resulting from *O*-methytransferas genes [10]. The chloroplasts (cps) play important functional roles in the photosynthesis, biosynthesis, and metabolism of starch and fatty acids throughout the plant’s life cycle [11]. Typically, the cp genomes of angiosperm are circulars, with a characteristic quadripartite structure that is comprised of two inverted repeats (IRs) and two single copy regions: a large single copy region (LSC) and a small single copy region (SSC). The genetic composition of cp genome in angiosperm is more or less conserved, containing 110 to 120 genes including protein-coding genes (PCGs), transfer RNA (tRNA) genes and ribosomal RNA (rRNA) genes. In spite of the generally high conservation of gene order and gene content, cp genomes in angiosperm have undergone size changes, structure rearrangement, contraction and expansion of IRs, and even pseudogenization due to adaptations, even within genera, to the host plants’ environments [12].

Here we report the sequence and structural analyses of the complete cp genome of *R. chinensis* var. *spontanea,* including analyses of the repeats and SSRs. Furthermore, we carried out comparative sequence analysis studies of cp sequences in the genus *Rosa*. This information will be useful for the conservation genetics of *R. chinensis* var. *spontanea,* as well as for the phylogenetic study of the genus *Rosa*. It might also benefit the genetics and breeding of modern roses.

## 2. Results and Discussion

### 2.1. Characteristics of Chloroplast Genome of R. chinensis var. spontanea

**Figure 1:**
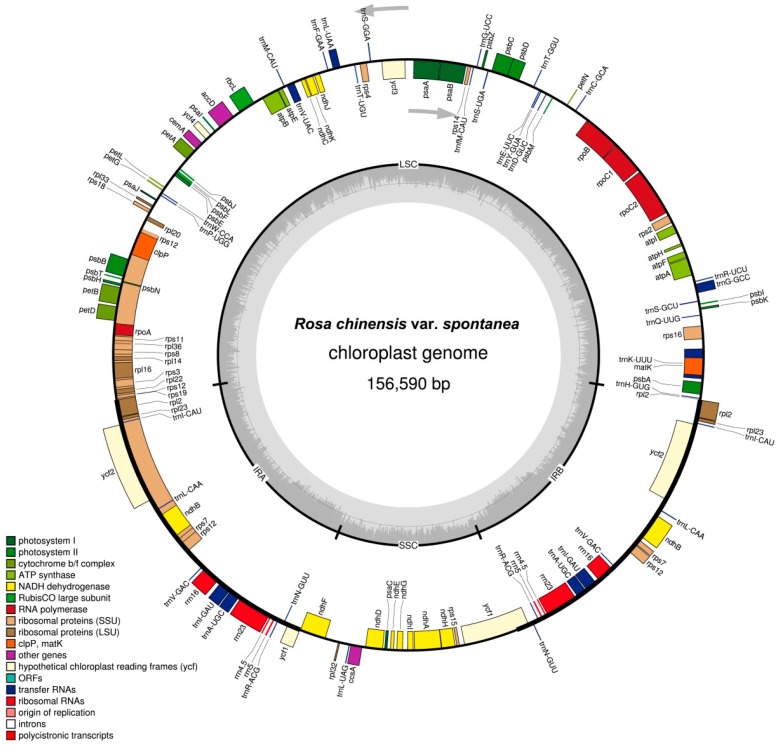
Chloroplast genome map of *Rosa chinensis* var. *spontanea*. Genes inside the circle are transcribed clockwise, and those outside are counterclockwise. Genes of different functions are color-coded. The darker gray in the inner circle shows the GC content, while the lighter gray shows the AT content.

The complete cp genome of *R. chinensis* var. *spontanea* represents a typical quadripartite circular molecule that is 156,590 bp in length. It is composed by a LSC region of 85,910 bp and a SSC region of 18,762 bp, separated by two IR regions of 25,959 bp (Table 1 and Figure 1). The GC content of the total cp DNA sequence is 37.2%, similar to that of *R. odorata* (Andr.) Sweet var. *gigantea* (Crép) Rehd. et Wils.(KF753637) [13], *R. praelucens* Byhouwer (MG450565) [14] and *R. roxburghii* Tratt. (KX768420). The GC content of the IR regions is 42.8%, while the LSC and SSC regions exhibit lower GC content (35.2% and 31.2%, respectively) (Table 1). The complete cp genome includes 57.8% coding sequences (50.2% PCGs, 1.8% tRNAs, and 5.8% rRNAs) and 42.2% non-coding sequences (11.8% introns and 30.4% intergenic spacers). Among PCGs, the AT content of the first, second, and third positions is 54.7%, 62.5%, and 69.7%, respectively (Table 1). This kind of bias towards a higher AT content at the third position of the codons is used to discriminate cp DNA from nuclear and mitochondrial DNA [15] and has been widely reported in other plant cp genomes [16,17,18].

The cp genome of *R. chinensis* var. *spontanea* contains 129 genes, including 84 PCGs, 37 tRNAs, and eight rRNAs (Table S1). Six PCGs (*ndhB*, *rpl2*, *rpl23*, *rps7, rps12* and *ycf2)*, four rRNAs (*rrn16*, *rrn23*, *rrn 4.5* and *rrn5)* and seven tRNAs (*trnA*-*UGC*, *trnI*-*CAU*, *trnI-GAU*, *trnL*-*CAA*, *trnN*-*GUU*, *trnR*-*ACG,* and *trnV-GAC*) within the IR regions are completely duplicated. The LSC region contains 62 PCGs and 22 tRNAs. The SSC region contains one tRNA and 12 PCGs. Additionally, 14 genes, namely *trnK*-*UUU*, *rps16*, *trnG*-*GCC*, *rpoC1*, *trnL*-*UAA*, *trnV*-*UAC*, *petB*, *rpl16*, *rp12*, *ndhB*, *trnI*-*GAU*, *trnA*-*UGC*, *ndhA*, and *petD*, contain one intron, whereas the *ycf3*, *rps12* and *clpP* genes contain two introns. Despite that, there are 17–20 group II introns within tRNA and protein-coding genes in land plant cp genomes [19], so far only the intron of *trnL* has been characterized as a group I intron in chloroplasts [20]. Thus, all these introns of *R. chinensis* var. *spontanea*, except the *trnL-UAA* intron, might be group II introns. The *rps12* gene is trans-spliced in the cp genome of *R. chinensis* var. *spontanea*. C-terminal exon 2 and 3 of *rps12* are located in the IR regions. Exon 1 is 28,259 bp downstream of the nearest copy of exons 2 and 3 while 72,017 bp away from the distal copy of exons 2 and 3 (Table S1). The *trnK-UUU* gene had the largest intron with a 2498 bp length, in which the *matK* gene was located. The *matK* gene encodes MatK, the maturase which is derived from reverse transcriptase and has been proved to be an essential splice factor for both the group I and group II introns [20,21].

Based on the sequences of PCGs and tRNAs, the frequency of codon usage of the cp genome of *R. chinensis* var. *spontanea* was estimated (Table 2). In total, 27,525 codons were found in all the coding sequences. Among these, leucine is the most frequent amino acid, representing 10.4% (2,871) of the total codons, while cysteine is the least frequent one with 1.2% (320) of the codons. A- and U-ending codons are common. Except for *trnL-CAA*, *trnS-GGA* and a stop codon (UAG), all types of preferred synonymous codons (RSCU > 1) ended with A or U.

### 2.2. Repeat and SSR Analysis

For the repeat structure analysis, 33 forward and five inverted repeats with a minimal repeat size of 20 bp were detected in the cp genome of *R. chinensis* var. *spontanea* (Table 3). Most of these repeats are between 20 and 30 bp. The longest forward repeat is 41 bp in length, located in the intergenic region between the genes *psbE* and *petL*. Most of the repeats were found in the LSC region. Among them, repeat No. 5 is related to *trnS-GCU* and *trnS-UGA* (Table 3). Repeat No. 7 is related to *trnG-GCU* and *trnG-UCC*. Repeat No. 13 is associated with *psa* genes. Six forward repeats were located in IR regions, including two repeats associated with *ycf2* genes and one repeat related to the *ndhB* gene. In addition, there were several repeat pairs with either repeated sequence located in a distinct region, e.g., each of the two sequences of repeat No. 16, 25, and 26 are located in the gene introns of LSC and SSC, respectively.

As chloroplast-specific SSRs are uniparentally inherited and exhibit a high level of intraspecific polymorphism, they are widely used in population genetics, species identification, evolutionary processes research of wild plants [22,23], and as markers for linkage map construction and the breeding of crop plants [24,25]. In total, 85 SSRs were identified in the cp genome of *R. chinensis* var. s*pontanea*, most of which were detected in the LSC region (Table 4). Among them, 55 (64.7%) are mononucleotide SSRs, ten (11.8%) are dinucleotide SSRs, seven (8.2%) are trinucleotide SSRs, 10 (11.8%) are tetranucleotide SSRs, one (1.2%) is a pentanucleotide SSR, and two (2.4%) are hexanucleotide SSRs. Only 22 SSRs are located in genes and the others are in the intergenic regions. Fifty two (94.5%) of the mononucleotide SSRs belong to the A/T type, which is consistent with the hypothesis that cp SSRs are generally composed of short polyadenine (poly A) or polythymine (poly T) repeats and rarely contain tandem guanine (G) or cytosine (C) repeats. These cp SSR markers can be used in the conservation genetics of *R. chinensis* var. *spontanea*, as well as and in both the linkage map construction and molecular-marker-assisted selection of modern roses.

### 2.3. Comparative Analysis of the Chloroplast Genomes of the Genus Rosa

The complete cp genome sequence of *R. chinensis* var. *spontanea* was compared to that of *R. odorata* var. *gigantea* [13], *R. roxburghii* (KX768420) and *R. praelucens* (MG450565) [14]. *Rosa chinensis* var. *spontanea* has the smallest cp genome with the smallest IR region (25,959 bp), while *R. praelucens* has the largest cp genome with the largest LSC, at 86,313 bp (Table S2). No significant differences were found in the sequence lengths of SSC among the four species. The main reason for the length differences in cp genomes of different rose species is the size variation of the LSC and IR regions (Table S2).

**Figure 2:**
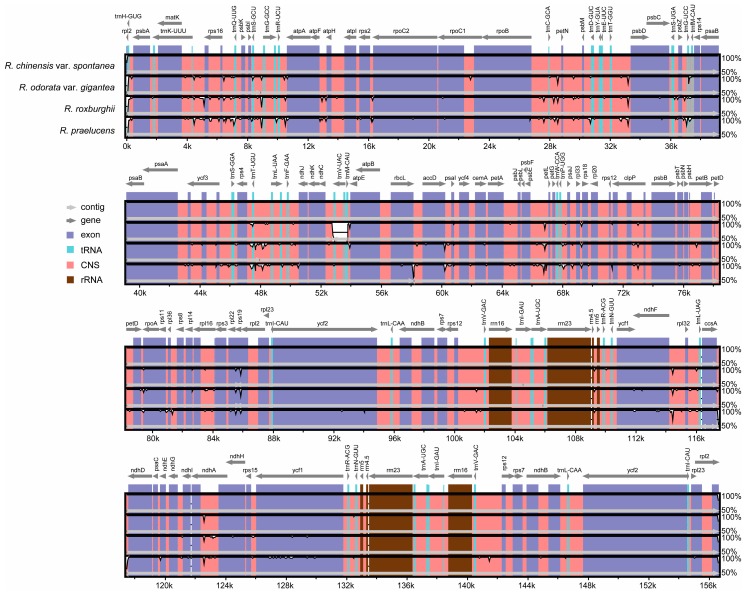
Complete chloroplast genome comparison of four rose species using the chloroplast genome of *R. chinensis* var. *spontanea* as a reference. The grey arrows and thick black lines above the alignment indicate the gene orientation. The *y*-axis represents the identity from 50% to 100%.

Sequence comparisons revealed that the LSC and the SSC regions were more divergent than the IR regions, and that higher divergence could be found in non-coding regions than in coding regions (Figure 2). Significant variations could be found in coding regions of some genes including *rps19* and *ycf1*. The highest divergence in non-coding regions was found in the intergenic regions of the *trnK-rps16*, *rps16-trnQ*, *trnS-trnG*, *trnR-atpA*, *atpF-atpH*, *rps2-rpoC2*, *rpoB-trnC*, *trnC-petN*, *trnT-psbD*, *psbZ-trnG*, *rps4-trnT*, *psbE-petL*, *trnP-psaJ*, *ndhF-rpl32*, and *ccsA-ndhD.* The introns of *rpl2, rps16, ndhA, trnV, clpP*, and *ndhA* were relatively highly divergent, too. These results might indicate that these regions evolve rapidly in the genus *Rosa*, as well as in other Rosaceae plants [26,27].

### 2.4. IR Contraction in the Chloroplast Genome of R. chinensis var. spontanea

**Figure 3:**
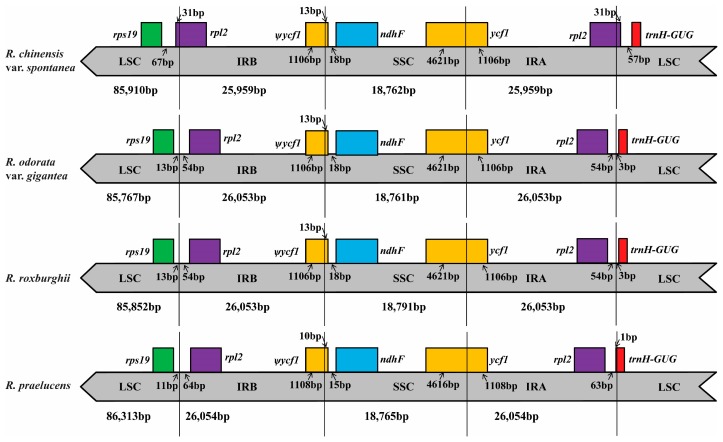
Comparison of the LSC, SSC and IR regions in chloroplast genomes of four species. Ψ: pseudogenes, →: distance from the edge.

Although IRs are the most conserved regions of the cp genomes, contraction and expansion at the borders of IR regions are common evolutionary events, and are hypothesized to be the main reason for size differences between cp genomes [28]. Detailed comparisons of the IR-SSC and IR-LSC boundaries among the cp genomes of the above four rose species were presented in Figure 3. IR regions are relatively highly conserved in the genus *Rosa*, but compared to other congeneric species, some position changes occurred in the IR/LSC regions of *R. chinensis* var. *Spontanea.* The *rpl2* gene in the cp genome shifted by 31 bp from IRb to LSC at the LSC/IRb border, and that gene also shifted by 31 bp from IRa to LSC at the IRa/LSC border, indicating the IR contraction in the cp genome of this species. This contraction is mainly caused by the fragment deletions in the intergenic regions of the *rps12-trnV*, *rrn4.5-rrn5*, and *trnR-trnN* genes, and leads to the relatively smaller size of its IR regions and consequently a smaller size of the cp genome (Figure 3, Table S2).

Generally, the IRa/LSC border is located between the *rpl2* and *trnH* genes in the rose family with *rpl2* in IRa and *trnH* in LSC [27], like in *R. roxburghii* and *R. odorata* var. *gigantea*. The *trnH* gene of *R. praelucens* extends only one bp from LSC to IRa, but its LSC region was much larger than that of other species (Table S2). One 505 bp insertion in the intergenic region between the genes *psbM* and *trnD* was detected according to the result of the MAFFT alignment. This large insertion leads to the largest LSC region of *R. praelucens* and thus the largest cp genome among these four rose species. The extraction and contraction of the IR region at the IR-SSC boundaries among these species were not significant. Accordingly, the extension and contraction of IR regions at the IR/LSC borders, along with the large insertion/deletion in the LSC region, might be the main reason for the cp genome size variation in the genus *Rosa*.

### 2.5. Phylogenetic Analysis

**Figure 4:**
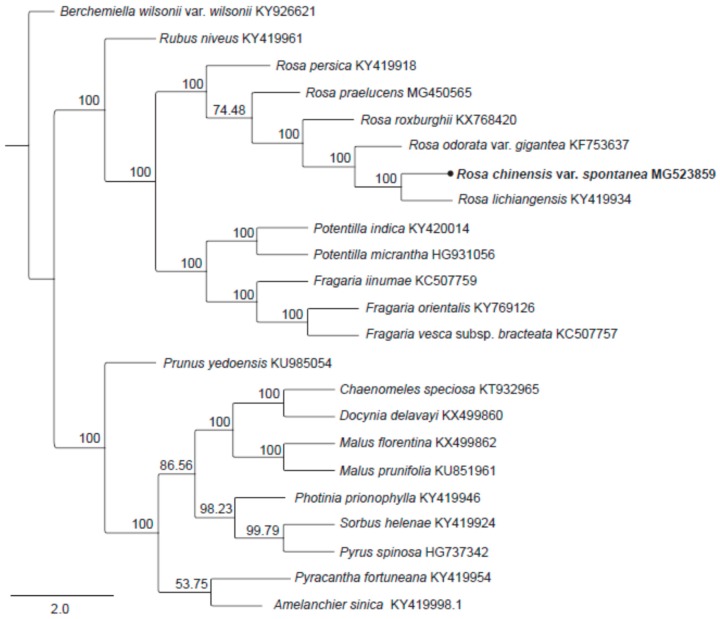
Phylogeny of 22 species within Rosaceae based on the ML analysis of the chloroplast genome’s LSC, SSC, and one-IR regions with *Berchemiella wilsonii* (Rhamnaceae) as the outgroup. The position of *R. chinensis* var. *spontanea* is shown in block letters.

There have been many attempts to reconstruct the phylogeny of the genus *Rosa*. Most of them suggested that the extant classification system was artificial [29,30] and that interspecies relationships within the genus remained ambiguous. The specific relationships within the sections *Chinenses* and *Synstylae* were still obscure due to limited sampling, low genetic variation of molecular markers, and complex evolutionary histories [31]. The availability of the complete cp genomes will provide additional informative data for the reconstruction of a robust phylogenetic model for the rose species. The phylogenetic tree (Figure 4) based on the LSC, SSC and one-IR regions in the cp genomes of 22 species from Rosaceae showed that species from Rosaceae were monophyletic and that the intra-family relationships were almost in compliance with that found by Zhang et al. [32]. Species from the genus *Rosa* formed a monophyletic clade with 100% support. The representative of the subgenus *Hulthemia*, *R. persica* Michx. [33,34]*,* was a sister to the clade composed by the other five rose species, supporting the subgenus position of *Hulthemia*. In the subgenus *Rosa*, *R. chinensis* var. *spontanea* from section *Chinenses* was sister to *R. lichiangensis* from section *Synstylae*, and then clustered with another species from section *Chinenses*, *R. odorata* var. *gigantean*, confirming that *R.* sections *Chinenses* and *Synstylae*, defined in the traditional taxonomic system, shared a more recent ancestor and could be merged as one section in the genus *Rosa* [30].

## 3. Materials and Methods

### 3.1. DNA Sequencing and Chloroplast Genome Assembly

Dry leaves of *R. chinensis* var. *spontanea* collected from Yichang of Hubei (111°10′ E, 30°47′ N, 400 m) were used to extract the total genomic DNA. A shotgun library was prepared and sequenced using the Illumina Hiseq 2000 (Illumina, CA, USA) at Novogene (Beijing, China). Approximately 3.68 Gb raw data of 150 bp paired-end reads were generated. The raw reads were filtered to obtain high-quality clean reads by using NGS QC Toolkit v2.3.3 with default parameters [35]. The cp genome was de novo assembled using the GetOrganelle pipeline (https://github.com/Kinggerm/GetOrganelle).

### 3.2. Gene Annotation and Sequence Analysis

The genome was automatically annotated by using the CpGAVAS pipeline [36]. The annotation was adjusted and confirmed by Geneious 8.1 [37]. Sequence data was deposited into GenBank under the accession number MG523859. The circular cp map of *R. chinensis* var. *spontanea* was generated by OGDRAW [38]. Codon usage analysis, calculation of relative synonymous codon usage values (RSCU), and measurement of AT content were carried out by using MEGA 6.06 [39].

### 3.3. Genome Comparison

MUMer [40] was used to perform pairwise sequence alignments of cp genomes. The mVISTA [41] program was applied to compare the complete cp genome of *R. chinensis* var. *spontanea* to the other published cp genomes of its congeneric species, i.e., *R. odorata* var. *gigantea*, *R. roxburghii* and *R. praelucens*, using the shuffle-LAGAN mode [42] and using the annotation of *R. chinensis* var. *spontanea* as reference. 

### 3.4. Repeats and Simple Sequence Repeats (SSRs)

REPuter [43] was used to find forward and inverted tandem repeats ≥ 20 bp with a minimum alignment score and maximum period size of 100 and 500, respectively. The minimum identity of repeats was limited to 85% (Hamming distance of 3). IMEx [44] was used to identify SSRs with the minimum repeat number set to 10, 5, 4, 3, 3 and 3 for mono-, di-, tri-, tetra-, penta- and hexanucleotides, respectively.

### 3.5. Phylogenetic Analysis

To identify the phylogenetic position of *R. chinensis* var. *spontanea* in *Rosa*, 21 published cp genomes of Rosaceae were used to construct a phylogeny tree, using *Berchemiella wilsonii* (C. K. Schneid.) Nakai (Rhamnaceae) as the outgroup. The LSC, SSC, and one-IR regions of the total 23 cp genomes were all aligned using MAFFT 7.308 [45]. The maximum likelihood (ML) tree was reconstructed by RAxML 8.2.11 [46] with the nucleotide substitution model of GTR + Gamma; node support was conducted by means of a bootstrap analysis with 1000 replicates.

## 4. Conclusions

In this study, we report and analyze the first complete cp genome of *R. chinensis* var. *spontanea*, one of the key ancestors of modern roses and a source for famous traditional Chinese medicines against female diseases. Compared to the cp genomes of other rose species, the cp genome of *R. chinensis* var. *spontanea* is the smallest, most likely due to the contraction of IR regions by 31 bps on each IR/LSC border. The cp genome of *R. chinensis* var. *spontanea* is rich in SSRs, which are valuable sources for developing new molecular markers. Our phylogenetic analysis showed that sampled species of the genus *Rosa* formed a monophyletic clade. *Rosa chinensis* var. s*pontanea* shared a more recent ancestor with *R. lichiangensis* of the section *Synstylae* than with *R. odorata* var. *gigantea* of the section *Chinenses*. This supported the hypothesis that, in the traditional taxonomic system, *Rosa* sections *Chinenses* and *Synstylae* were closely related and could be merged to a single section within the genus *Rosa*. This information will be useful for the conservation genetics of *R. chinensis* var. *spontanea* and the phylogenetic study of genus *Rosa*, and might also facilitate the genetics and breeding of modern roses.

## Figures and Tables

**Table 1:** Base composition in the chloroplast genome of *Rosa chinensis* var. *spontanea.*

Region		A	T (U)	G	C	Length
LSC		31.7	33.1	17.2	18.0	85,910
SSC		34.4	34.3	15.1	16.3	18,762
IRB		28.7	28.5	22.2	20.6	25,959
IRA		28.7	28.5	22.2	20.6	25,959
Total		31.0	31.8	18.6	18.7	156,590
	PCGs	30.6	31.4	20.3	17.7	79,773
	1st position	30.7	24	26.9	18.7	26,591
	2nd position	29.5	33	17.7	20.2	26,591
	3rd position	31.7	38	16.4	14.1	26,591

PCGs: protein-coding genes.

**Table 2:** Condon-anticodon recognition patterns and codon usage of the *Rosa chinensis* var. *spontanea* chloroplast genome.

Amino Acid	Codon	Count	RSCU	tRNA	Amino Acid	Codon	Count	RSCU	tRNA
Phe	UUU	1015	1.3		Ser	UCU	580	1.62	
Phe	UUC	545	0.7	*trnF-GAA*	Ser	UCC	370	1.03	*trnS-GGA*
Leu	UUA	897	1.87		Ser	UCA	406	1.13	*trnS-UGA*
Leu	UUG	580	1.21	*trnL-CAA*	Ser	UCG	222	0.62	
Leu	CUU	595	1.24		Pro	CCU	424	1.45	
Leu	CUC	217	0.45		Pro	CCC	241	0.82	
Leu	CUA	380	0.79		Pro	CCA	320	1.09	*trnP-UGG*
Leu	CUG	202	0.42		Pro	CCG	187	0.64	
Ile	AUU	1136	1.48		Thr	ACU	542	1.55	
Ile	AUC	451	0.59	*trnI-CAU*	Thr	ACC	269	0.77	*trnT-GGU*
Ile	AUA	716	0.93		Thr	ACA	418	1.19	*trnT-UGU*
Met	AUG	635	1	*trnM-CAU*	Thr	ACG	171	0.49	
Val	GUU	550	1.44		Ala	GCU	645	1.76	
Val	GUC	193	0.5	*trnV-GAC*	Ala	GCC	244	0.67	
Val	GUA	567	1.48		Ala	GCA	391	1.07	
Val	GUG	223	0.58		Ala	GCG	183	0.5	
Tyr	UAU	798	1.6		Cys	UGU	237	1.48	
Tyr	UAC	198	0.4	*trnY-GUA*	Cys	UGC	83	0.52	*trnC-GCA*
stop	UAA	59	1.38		stop	UGA	21	0.49	
stop	UAG	48	1.13		Trp	UGG	484	1	*trnW-CCA*
His	CAU	476	1.49		Arg	CGU	362	1.28	*trnR-ACG*
His	CAC	161	0.51	*trnH-GUG*	Arg	CGC	120	0.43	
Gln	CAA	734	1.51	*trnQ-UUG*	Arg	CGA	385	1.36	
Gln	CAG	236	0.49		Arg	CGG	144	0.51	
Asn	AAU	1003	1.52		Ser	AGU	420	1.17	
Asn	AAC	317	0.48		Ser	AGC	156	0.43	*trnS-GCU*
Lys	AAA	1082	1.48		Arg	AGA	488	1.73	*trnR-UCU*
Lys	AAG	385	0.52		Arg	AGG	194	0.69	
Asp	GAU	890	1.62		Gly	GGU	612	1.3	
Asp	GAC	207	0.38	*trnD-GUC*	Gly	GGC	209	0.44	*trnG-GCC*
Glu	GAA	1052	1.46	*trnE-UUC*	Gly	GGA	694	1.48	
Glu	GAG	390	0.54		Gly	GGG	365	0.78	

RSCU: Relative Synonymous Codon Usage.

**Table 3:** Repeat sequences in the *Rosa chinensis* var. *spontanea* chloroplast genome.

ID	Repeat Start 1	Type	Size (bp)	Repeat Start 2	Mismatch (bp)	E-Value	Gene	Region
1	4426	F	29	45,071	−2	8.74 × 10^−^^5^	IGS; *ycf3*(intron)	LSC
2	4427	F	30	4428	−3	6.56 × 10^−^^4^	IGS	LSC
3	4428	F	28	45,072	−3	8.47 × 10^−^^3^	IGS	LSC
4	4432	F	26	45,072	−2	4.48 × 10^−^^3^	IGS	LSC
5	8329	F	29	36,077	−2	8.74 × 10^−^^5^	*trnS-GCU*; *trnS-UGA*	LSC
6	8873	F	20	8895	0	6.27 × 10^−^^3^	IGS	LSC
7	9804	F	27	37,135	−1	3.10 × 10^−^^5^	*trnG-GCU*; *trnG-UCC*	LSC
8	13,510	F	20	89,606	0	6.27 × 10^−^^3^	IGS; *ycf2*	LSC; IRa
9	14,236	F	20	29,560	0	6.27 × 10^−^^3^	IGS	LSC
10	27,619	F	24	27,643	0	2.45 × 10^−^^5^	IGS	LSC
11	29,555	F	24	29,556	−1	1.76 × 10^−^^3^	IGS	LSC
12	33,157	F	20	33,177	0	6.27 × 10^−^^3^	IGS	LSC
13	39,390	F	30	41,614	−3	6.56 × 10^−^^4^	*psaB; psaA*	LSC
14	42,625	F	25	147,248	−1	4.59 × 10^−^^4^	IGS	LSC; IRb
15	44,406	F	39	100,262	0	2.28 × 10^−^^14^	*ycf3*(intron); IGS	LSC; IRa
16	44,406	F	38	122,332	0	9.13 × 10^−^^14^	*ycf3*(intron); *ndhA*(intron)	LSC; SSC
17	45,075	F	24	142,008	−1	1.76 × 10^−^^3^	*ycf3*(intron); IGS	LSC; IRb
18	47,622	F	25	47,645	0	6.13 × 10^−^^6^	IGS	LSC
19	58,656	F	34	58,687	0	2.34 × 10^−^^11^	IGS	LSC
20	66,712	F	41	66,752	0	1.43 × 10^−1^^5^	IGS	LSC
21	66,939	F	20	66,958	0	6.27 × 10^−^^3^	IGS	LSC
22	68,033	F	21	68,052	0	1.57 × 10^−^^3^	IGS	LSC
23	71,232	F	20	84,928	0	6.27 × 10^−^^3^	IGS	LSC
24	80,953	F	27	80,966	−2	1.21 × 10^−^^3^	IGS	LSC
25	83,166	F	29	122,320	−3	2.36 × 10^−^^3^	*rpl16*(intron); *ndhA*(intron)	LSC;SSC
26	83,172	F	28	122,326	−3	8.47 × 10^−^^3^	*rpl16*(intron); *ndhA*(intron)	LSC;SSC
27	90,610	F	29	90,631	−2	8.74 × 10^−^^5^	*ycf2*	IRa
28	97,630	F	31	144,839	−3	1.81 × 10^−^^4^	*ndhB*(intron)	IRa; IRb
29	100,260	F	40	122,330	0	5.70 × 10^−1^^5^	IGS; *ndhA*(intron)	IRa; SSC
30	101,012	F	23	101,033	0	9.80 × 10^−^^5^	IGS	IRa
31	141,437	F	30	141,458	−2	2.34 × 10^−^^5^	IGS	IRb
32	141,444	F	23	141,465	0	9.80 × 10^−^^5^	IGS	IRb
33	151,840	F	29	151,861	−2	8.74 × 10^−^^5^	*ycf2*	IRb
34	6406	I	20	71,231	0	6.27 × 10^−^^3^	IGS	LSC
35	6408	I	24	71,228	−1	1.76 × 10^−^^3^	IGS	LSC
36	8622	I	26	45,073	−2	4.48 × 10^−^^3^	IGS	LSC
37	8625	I	23	45,077	−1	6.76 × 10^−^^3^	IGS	LSC
38	71,232	I	20	84,930	0	6.27 × 10^−^^3^	IGS	LSC

F: Forward; I: Inverted; IGS: intergenic space.

**Table 4:** Simple sequence repeats (SSRs) in the *Rosa chinensis* var. *spontanea* chloroplast genome.

ID	Repeat Motif	Length (bp)	Start	End	Region	Gene	ID	Repeat Motif	Length (bp)	Start	End	Region	Gene
1	(A)11	11	279	289	LSC		44	(TTTA)4	12	50,468	50,479	LSC	
2	(T)11	11	4108	4118	LSC		45	(TA)5	10	52,742	52,751	LSC	
3	(A)19	19	4428	4446	LSC		46	(T)10	10	55,811	55,820	LSC	*atpB*
4	(A)10	10	4449	4458	LSC		47	(AAAT)3	12	55,911	55,922	LSC	
5	(A)10	10	4887	4896	LSC		48	(TAAT)3	12	58,366	58,377	LSC	
6	(T)10	10	5023	5032	LSC		49	(T)14	14	60,810	60,823	LSC	
7	(TATAT)3	15	6102	6116	LSC	*rps16*	50	(TC)5	10	62,280	62,289	LSC	*cemA*
8	(T)17	17	6407	6423	LSC		51	(T)11	11	64,513	64,523	LSC	
9	(AATA)3	12	6525	6536	LSC		52	(T)10	10	69,689	69,698	LSC	
10	(AG)5	10	6755	6764	LSC		53	(A)16	16	69,739	69,754	LSC	
11	(A)11	11	6945	6955	LSC		54	(T)18	18	71,235	71,252	LSC	
12	(TAA)4	12	8257	8268	LSC		55	(T)15	15	71,933	71,947	LSC	*clpP*
13	(A)10	10	8639	8648	LSC		56	(A)10	10	72,733	72,742	LSC	*clpP*
14	(AT)6	12	10,093	10,104	LSC		57	(AT)6	12	73,632	73,643	LSC	
15	(TAT)4	12	10,343	10,354	LSC		58	(A)12	12	79,231	79,242	LSC	
16	(T)11	11	12,157	12,167	LSC		59	(A)14	14	79,393	79,406	LSC	
17	(T)10	10	12,915	12,924	LSC		60	(T)10	10	79,429	79,438	LSC	*rpoA*
18	(A)10	10	13,184	13,193	LSC		61	(ATGT)3	12	79,529	79,540	LSC	*rpoA*
19	(C)10	10	14,237	14,246	LSC		62	(T)11	11	81,586	81,596	LSC	
20	(T)10	10	14,247	14,256	LSC		63	(A)10	10	82,641	82,650	LSC	
21	(T)11	11	18,361	18,371	LSC	*rpoC2*	64	(A)12	12	83,422	83,433	LSC	*rpl16*
22	(TA)5	10	19,730	19,739	LSC	*rpoC2*	65	(A)11	11	83,498	83,508	LSC	*rpl16*
23	(T)10	10	26,080	26,089	LSC	*rpoB*	66	(T)18	18	84,931	84,948	LSC	
24	(T)12	12	28,925	28,936	LSC		67	(TAT)4	12	86,619	86,630	IRa	*rpl2*
25	(C)15	15	29,556	29,570	LSC		68	(TAGAAG)3	18	93,987	94,004	IRa	*ycf2*
26	(T)10	10	29,571	29,580	LSC		69	(T)11	11	101,618	101,628	IRa	
27	(AAT)4	12	30,504	30,515	LSC		70	(AGGT)3	12	107,843	107,854	IRa	*rrn23*
28	(T)14	14	30,519	30,532	LSC		71	(TATT)3	12	110,028	110,039	IRa	
29	(A)10	10	30,666	30,675	LSC		72	(TGT)4	12	111,869	111,880	SSC	
30	(TA)5	10	36,313	36,322	LSC		73	(T)10	10	115,507	115,516	SSC	
31	(T)11	11	36,473	36,483	LSC		74	(TAA)4	12	115,558	115,569	SSC	
32	(AT)5	12	37,070	37,079	LSC		75	(A)13	13	115,612	115,624	SSC	
33	(C)13	13	37,303	37,315	LSC		76	(T)10	10	120,845	120,854	SSC	
34	(A)11	11	37,316	37,326	LSC		77	(AT)7	14	121,678	121,691	SSC	
35	(AT)5	10	43,682	43,691	LSC	*ycf3*	78	(A)16	16	122,551	122,566	SSC	*ndhA*
36	(A)15	15	45,073	45,087	LSC	*ycf3*	79	(T)15	15	122,804	122,818	SSC	*ndhA*
37	(A)10	10	45,392	45,401	LSC		80	(T)10	10	129,830	129,839	SSC	*ycf1*
38	(T)10	10	45,931	45,940	LSC		81	(ATAA)3	12	132,463	132,474	IRb	
39	(A)11	11	47,296	47,306	LSC		82	(CTAC)3	12	134,645	134,656	IRb	*rrn23*
40	(TAAT)3	12	48,112	48,123	LSC		83	(A)11	11	140,873	140,883	IRb	
41	(T)14	14	48,306	48,319	LSC		84	(CTTCTA)3	18	148,497	148,514	IRb	*ycf2*
42	(A)12	12	48,420	48,431	LSC		85	(ATA)4	12	155,871	155,882	IRb	
43	(TA)5	10	48,500	48,509	LSC								

## Supplementary Materials

Supplementary materials are available online.
