# Supplementary material for: The Complete Chloroplast Genome of a Key Ancestor of Modern Roses, Rosa chinensis var. spontanea, and a Comparison with Congeneric Species
Source: Molecules. 2018 Feb 12;23(2):389. doi: 10.3390/molecules23020389 (PMC6017658; doi:10.3390/molecules23020389)
Supplement: Supplementary file 1 [file molecules-23-00389-s001.pdf]

Table S1 Genes encoded in the chloroplast genome of *Rosa chinensis* var. *spontanea*

Gene	Minimum	Maximum	Length(bp)	Direction	Exon I (bp)	Intron I (bp)	Exon II (bp)	Intron II (bp)	Exon III (bp)
trnH-GUG	89	162	74	reverse					
psbA	450	1,511	1,062	reverse					
trnK-UUU	1,776	4,345	2,570	reverse	35	2,498	37		
matK	2,069	3,580	1,512	reverse					
rps16	5,149	6,292	1,144	reverse	228	874	42		
trnQ-UUG	7,143	7,214	72	reverse					
psbK	7,634	7,819	186	forward					
psbI	8,058	8,168	111	forward					
trnS-GCU	8,333	8,420	88	reverse					
trnG-GCC	9,066	9,831	766	forward	23	695	48		
trnR-UCU	10,011	10,082	72	forward					
atpA	10,624	12,147	1,524	reverse					
atpF	12,200	12,754	555	reverse					
atpH	13,242	13,487	246	reverse					
atpI	14,420	15,163	744	reverse					
rps2	15,389	16,099	711	reverse					
rpoC2	16,340	20,494	4,155	reverse					
rpoC1	20,672	23,481	2,810	reverse	1,611	764	435		
rpoB	23,513	26,725	3,213	reverse					
trnC-GCA	27,944	28,014	71	forward					
petN	28,791	28,880	90	forward					
psbM	30,158	30,262	105	reverse					
trnD-GUC	30,785	30,858	74	reverse					
trnY-GUA	31,279	31,362	84	reverse					
trnE-UUC	31,422	31,494	73	reverse					
trnT-GGU	32,010	32,081	72	forward					
psbD	33,410	34,471	1,062	forward					
psbC	34,419	35,840	1,422	forward					
trnS-UGA	36,081	36,173	93	reverse					
psbZ	36,555	36,743	189	forward					
trnG-UCC	37,093	37,163	71	forward					
trnfM-CAU	37,364	37,437	74	reverse					
rps14	37,600	37,902	303	reverse					
psaB	38,029	40,233	2,205	reverse					
psaA	40,259	42,511	2,253	reverse					
ycf3	43,222	45,235	2,014	reverse	153	766	228	741	126
trnS-GGA	46,113	46,199	87	forward					
rps4	46,492	47,097	606	reverse					
trnT-UGU	47,496	47,568	73	reverse					
trnL-UAA	48,749	49,379	631	forward	37	544	50		
trnF-GAA	49,785	49,857	73	forward					
ndhJ	50,588	51,064	477	reverse					
ndhK	51,187	51,867	681	reverse					
ndhC	51,911	52,273	363	reverse					
trnV-UAC	52,876	53,547	672	reverse	37	596	39		
trnM-CAU	53,715	53,787	73	forward					
atpE	53,964	54,365	402	reverse					
atpB	54,388	55,866	1,479	reverse					
rbcL	56,649	58,076	1,428	forward					
accD	58,740	60,203	1,464	forward					

psaI	60,671	60,784	114	forward					
ycf4	61,208	61,762	555	forward					
cemA	62,213	62,902	690	forward					
petA	63,111	64,073	963	forward					
psbJ	65,041	65,163	123	reverse					
psbL	65,300	65,416	117	reverse					
psbF	65,439	65,558	120	reverse					
psbE	65,568	65,819	252	reverse					
petL	67,079	67,174	96	forward					
petG	67,350	67,463	114	forward					
trnW-CCA	67,588	67,661	74	reverse					
trnP-UGG	67,806	67,879	74	reverse					
psaJ	68,317	68,451	135	forward					
rpl33	68,911	69,111	201	forward					
rps18	69,324	69,629	306	forward					
rpl20	69,923	70,276	354	reverse					
*rps12**	71,057	143,071	908	mixed	114		232	536	26
*rps12**	71,057	100,223	908	reverse	26	537	232		114
clpP	71,372	73,447	2,076	reverse					
psbB	73,899	75,425	1,527	forward					
psbT	75,611	75,712	102	forward					
psbN	75,787	75,918	132	reverse					
psbH	76,037	76,261	225	forward					
petB	76,384	77,819	1,436	forward	6	788	642		
petD	78,013	79,215	1,203	forward					
rpoA	79,416	80,411	996	reverse					
rps11	80,471	80,887	417	reverse					
rpl36	81,046	81,159	114	reverse					
rps8	81,640	82,044	405	reverse					
rpl14	82,230	82,598	369	reverse					
rpl16	82,737	84,120	1,384	reverse	399	976	9		
rps3	84,230	84,886	657	reverse					
rpl22	85,022	85,441	420	reverse					
rps19	85,534	85,812	279	reverse					
rpl2	85,880	87,385	1,506	reverse	434	681	391		
rpl23	87,404	87,685	282	reverse					
trnI-CAU	87,851	87,924	74	reverse					
ycf2	88,013	94,852	6,840	forward					
trnL-CAA	95,807	95,887	81	reverse					
ndhB	96,388	98,596	2,209	reverse	756	676	777		
rps7	98,910	99,377	468	reverse					
trnV-GAC	101,966	102,037	72	forward					
rrn16	102,265	103,755	1,491	forward					
trnI-GAU	104,053	105,078	1,026	forward	42	949	35		
trnA-UGC	105,143	106,027	885	forward	38	812	35		
rrn23	106,191	108,998	2,808	forward					
rrn4.5	109,097	109,199	103	forward					
rrn5	109,448	109,568	121	forward					
trnR-ACG	109,825	109,898	74	forward					
trnN-GUU	110,365	110,436	72	reverse					
ndhF	111,901	114,144	2,244	reverse					
rpl32	115,272	115,430	159	forward					
trnL-UAG	116,194	116,273	80	forward					

ccsA	116,368	117,333	966	forward			
ndhD	117,587	119,089	1,503	reverse			
psaC	119,214	119,459	246	reverse			
ndhE	119,702	120,007	306	reverse			
ndhG	120,247	120,777	531	reverse			
ndhI	121,167	121,670	504	reverse			
ndhA	121,754	124,062	2,309	reverse	540	1,217	552
ndhH	124,064	125,245	1,182	reverse			
rps15	125,349	125,621	273	reverse			
ycf1	126,011	131,737	5,727	reverse			
trnN-GUU	132,065	132,136	72	forward			
trnR-ACG	132,603	132,676	74	reverse			
rrn5	132,933	133,053	121	reverse			
rrn4.5	133,302	133,404	103	reverse			
rrn23	133,503	136,310	2,808	reverse			
trnA-UGC	136,474	137,358	885	reverse	35	812	38
trnI-GAU	137,423	138,448	1,026	reverse	35	949	42
rrn16	138,746	140,236	1,491	reverse			
trnV-GAC	140,464	140,535	72	reverse			
rps7	143,124	143,591	468	forward			
ndhB	143,905	146,113	2,209	forward	776	676	757
trnL-CAA	146,614	146,694	81	forward			
ycf2	147,649	154,488	6,840	reverse			
trnI-CAU	154,577	154,650	74	forward			
rpl23	154,816	155,097	282	forward			
rpl2	<1	>156590	1506	forward	391	681	404

* The *rps12* gene is a trans-spliced gene with the 50 end located in the LSC region and the duplicated 30 ends in the IR region.

Table S2 Size comparison of complete cp genomes of four rose species

**Species**	**LSC**	**IRB**	**SSC**	**IRA**	**Total**
Rosa chinensis var. *spontanea*	85910	25959	18762	25959	156590
Rosa odorata var. *gigantea*	85767	26053	18761	26053	156634
Rosa roxburghii	85852	26053	18791	26053	156749
Rosa praelucens	86313	26054	18765	26054	157186
